# Brain-derived neurotrophic factor (BDNF) as a potential marker of endometriosis: a systematic review and meta-analysis

**DOI:** 10.1186/s12905-023-02877-0

**Published:** 2024-01-13

**Authors:** Kyana Jafarabady, Arman Shafiee, Razman Arabzadeh Bahri, Ida Mohammadi, Mohammad javad Amini, Shahryar Rajai, Diba Akbarzadeh, Faeze Soltani Abhari, Emad Movahed, Soraya Parvari, Mahmood Bakhtiyari

**Affiliations:** 1 Student Research Committee, School of Medicine, Alborz University of Medical Sciences, Karaj, Iran; 2 Department of Psychiatry and Mental Health, Alborz University of Medical Sciences, Karaj, Iran; 3 School of Medicine, Tehran University of Medical Sciences, Tehran, Iran; 4 School of Medicine, Shahid Beheshti University of Medical Sciences, Tehran, Iran; 5 Department of Anatomical Sciences, School of Medicine, Alborz University of Medical Sciences, Karaj, Iran; 6 Non-Communicable Diseases Research Center, Alborz University of Medical Sciences, Karaj, Iran; 7 Department of Community Medicine and Epidemiology, Alborz University of Medical Sciences, Karaj, Iran; 8 Dental Reaserch Center, Faculty of Dentistry, Islamic Azad University of Medical Sciences, Tehran, Iran

**Keywords:** Endometriosis, Brain-derived neurotrophic factor, BDNF

## Abstract

**Background:**

The existing literature on the association between BDNF protein levels and endometriosis presents inconsistent findings. This systematic review and meta-analysis aim to synthesize the available evidence and evaluate the possible relationship between BDNF protein levels and endometriosis.

**Methods:**

Electronic databases (PubMed, Embase, Scopus, PsycINFO, and Web of Science) were used to conduct a comprehensive literature search from inception to June 2023. The search strategy included relevant keywords and medical subject headings (MeSH) terms related to BDNF, endometriosis, and protein levels. A random-effects model was used for the meta-analysis, and to explore heterogeneity subgroup analyses were performed. funnel plots and statistical tests were used for assessing the publication bias.

**Results:**

A total of 12 studies were included. The pooled standardized mean difference (SMD) of BDNF levels between women with endometriosis and controls was 0.87 (95% confidence interval [CI] 0.34 to 1.39, *p* = 0.001; I2 = 93%). The results showed that blood levels of BDNF are significantly higher in endometriosis patients (SMD: 1.13 95% CI 0.54 to 1.73, *p* = 0.0002; I2 = 93%). No significant publication bias was observed based on the results of Egger’s regression test ((*p* = 0.15).

**Conclusion:**

This study revealed a significant difference between patients diagnosed with endometriosis and healthy control in the level of BDNF. The results indicate that women with endometriosis have higher levels of BDNF. Further studies are needed to be undertaken to investigate the role of BDNF in endometriosis pathophysiology and the diagnostic value of BDNF in endometriosis.

**Supplementary Information:**

The online version contains supplementary material available at 10.1186/s12905-023-02877-0.

## Introduction

Endometriosis is a chronic gynecological disorder characterized by the presence of endometrial-like tissue outside the uterus, most commonly in the pelvic cavity [1]. It affects approximately 10% of women of reproductive age and is associated with debilitating symptoms such as pelvic pain, dysmenorrhea, dyspareunia, and infertility [1]. The pathogenesis of endometriosis remains poorly understood, and there is a need for reliable biomarkers that can aid in its diagnosis and management [2].

Brain-derived neurotrophic factor (BDNF) is a neurotrophin that plays a crucial role in the development, survival, and plasticity of neurons in the central nervous system [3]. It has been implicated in various physiological processes, including neuronal growth, synaptic plasticity, and pain modulation [3, 4]. BDNF is primarily synthesized in the brain, but emerging evidence suggests that it is also expressed in peripheral tissues, including the reproductive system [5].

Recent studies have proposed a potential association between BDNF and endometriosis, highlighting BDNF as a promising candidate biomarker for this condition [6, 7]. Elevated levels of BDNF have been reported in the peritoneal fluid, serum, and endometrial tissue of women with endometriosis compared to healthy controls [8–10]. These findings suggest that BDNF may be involved in the pathogenesis of endometriosis and could potentially serve as a diagnostic or prognostic marker [7, 11]. However, the existing literature on the association between BDNF and endometriosis is still limited and characterized by inconsistencies in findings. Therefore, a comprehensive evaluation of the available evidence is warranted to clarify the role of BDNF in endometriosis. The aim of this systematic review and meta-analysis is to evaluate the existing evidence on the association between BDNF levels and endometriosis.

## Methods

The Preferred Reporting Items for Systematic Reviews and Meta-Analyses (PRISMA) guidelines was followed for conducting the present study. More details about PRISMA can be found in Supplementary File Table 1. The protocol of this study is registered in PROSPERO with the code CRD42023439147.

**Table 1:** Characteristics of the included studies

Author	Country	Year	Endometriosis type	Endometriosis stage	BDNF source	Age	Sample size (case/control)	Quality
de Arellano	Germany	2013	Peritoneal endometriotic lesions	NA	Peritoneal fluid	NA	40 (20/20)	Fair
Bucci	Italy	2011	NA	Stages 1 and 2	Plasma	case = 28.36 ± 3.9 control = 26.81 ± 4.53	22 (11/11)	Fair
Browne	USA	2012	NA	NA	Eutopic endometrial biopsy	case = 34 ± 7 control = 34 ± 6	33 (18/15)	Good
Herranz-Blanco	Spain	2023	superficial peritoneal lesions = 54 (39.7%) ovarian endometriomas = 26 (19.1%) deep infiltrating endometriosis = 29 (21.3%) deep infiltrating endometriosis and ovarian endometriomas = 25 (18.4%) Unclassified = 2 (1.5%)	rASRM classification I–II = 68 (50%) III–IV = 68 (50%)	serum samples	case = 35.6 ± 6.42 control = 33.5 ± 5.96	204 (136/68)	Fair
Giannini	Italy	2010	NA	stage I and II	plasma and follicular fluid	case = 29.8 ± 4.13 control = 27.7 ± 4.7	56 (26/30)	Poor
Dwiningsih	Indonesia	2022	Ovarian endometriosis n = 32 (88.9) Peritoneal endometriosis n = 4 (11.1)	rASRM classification I = 3 (8.3) II = 1 (2.8) III = 11 (30.6) IV = 21 (58.3)	Serum	case = 31.47 ± 6.5 control = 38.14 ± 4.4	50 (36/14)	Good
Ding	People’s Republic of China	2017	Ovarian endometrioma	Revised American Fertility Society scoring system I–II = 32 (53.3%) III–IV = 28 (46.7%)	Serum and peritoneal fluid	case = 35.3 ± 0.9 control = 35.6 ± 1.4	98 (60/38)	Good
Yu	USA	2023	NA	NA	Peritoneal fluid	Cases = 38.0 ± 6.0 Control = 43.0 ± 4.5	40 (14/26)	Good
Wessels	Canada	2016	NA	I: 10 II: 9 III: 10 IV: 64	Plasma	Cases = 34.7 ± 7.0 Control = 29.9 ± 8.5	104 (68/36)	Fair
Stefani	Brazil	2019	NA	NA	serum	NA	53 (36/17)	Fair
Perricos	Austria	2018	22 superficial peritoneal 3 deep infiltrating 12 endometrioma 32 combination of two 7 combination of three	I:21 II: 14 III: 20 IV: 20	serum	Cases = 33.7 ± 6.04 Controls = 34.8 ± 6.9	128 (77/51)	Good
Liang	China	2020	NA	NA	serum	NA	157 (82/75)	Poor

### Search strategy

A systematic search was performed in four international bibliometric databases, including Scopus, Embase, PubMed, and Web of Science from the inception up to 12 June 2023, with the goal of identifying any published article which evaluated the altered levels of BDNF in endometriosis. Regarding our systematic search strategy, we categorized the keywords into two different groups, including the endometriosis group and the BDNF group. In the endometriosis group, we used any possible keyword related to endometriosis, including endometriosis, adenomyosis, or abnormal uterine tissue. In the BDNF group, we used all possible keywords related to BDNF, such as BDNF, brain-derived neurotrophic factor, or brain-derived neurotrophic factor. We used “OR” between the keywords in each group and utilized “AND” between the groups. Supplementary Table 2 represents the search string for each database in detail.

### Eligibility criteria

We included studies that evaluated the levels of BDNF in endometriosis using enzyme-linked immunoassays (ELISA) or any other methods. The exclusion criteria included animal studies, in-vitro studies, meta-analyses, review articles, letters to editors, case reports, and congress abstracts. We did not impose any language restriction regarding the original language of the identified articles.

### Data extraction and quality assessment

The initial screening of the identified studies, based on their titles and abstracts was performed by two independent reviewers, in order to exclude irrelevant studies. Then, the full texts of the remained articles were evaluated for extracting their data. Two independent reviewers performed the data extraction, based on an Excel sheet, containing the first author’s names, country of origin, year of publication, type of endometriosis, the stage of the endometriosis, source of the BDNF, age of the patients, and sample sizes of the studies. Moreover, two independent reviewers assessed the quality of the included studies, using Newcastle-Ottawa Scale (NOS) tool.

### Data synthesis and meta-analysis

The meta-analysis utilized a random-effects model to determine the combined effect size and evaluate its statistical significance. The standardized mean difference (SMD) and its corresponding 95% confidence intervals (95% CIs) were employed to present the pooled effect sizes. Sensitivity analysis was performed by including only the studies that assessed blood levels of BDNF. Assessment of publication bias was conducted through the implementation of funnel plots and Egger’s regression test.

## Results

### Study selection

A systematic search of electronic databases yielded a total of 192 articles. After removing duplicates and applying the inclusion and exclusion criteria which was done by two reviewers (A.S & S.R), a final set of 12 articles were included in this systematic review and meta-analysis [6, 8–18] The characteristic information of included studies is in Table 1. The inclusion criteria were as follows: (1) patient population: women of reproductive age after being diagnosed with endometriosis; (2) Intervention: evaluating level of BDNF in serum or plasma; (3) Comparison: healthy women ; (4) Outcome: impact on the BDNF level; (5) Setting/Time: All and (6) study design: randomized controlled trial, retrospective studies, and prospective studies. Studies that were conducted on animals or have not met our inclusion criteria or were designed as case reports, case series, and non-English articles were excluded.

**Fig. 1:**
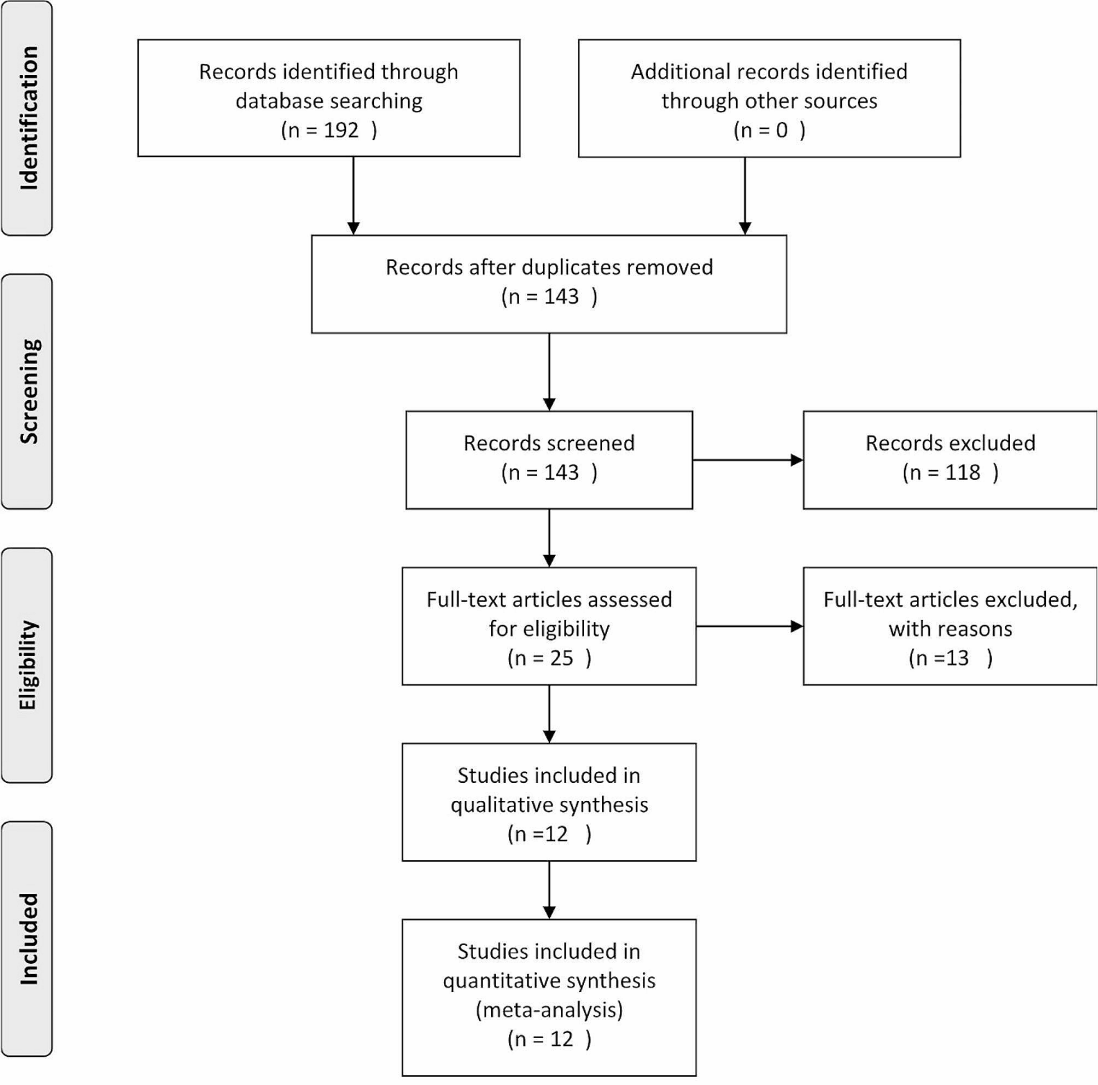
PRISMA flow diagram

The selection process is illustrated in Fig. 1.

### Characteristics of included studies

#### Quality assessment

The quality assessment of the included studies was performed using the Newcastle-Ottawa Scale (NOS) for observational studies (Table 2). The overall quality of the studies ranged from moderate to high, with most studies scoring 6 or higher on the NOS. Only two studies had poor quality [8, 14].

**Table 2:** Results of quality assessments

Author	Year	Is the case definition adequate?	Representativeness of the cases	Selection of Controls	Definition of Controls	Comparability	Ascertainment of exposure	Same method of ascertainment for cases and controls	Non-Response rate	Total
Maria Luisa Barcena de Arellano	2013	1	1	0	1	0	1	1	1	Fair
Fiorella Bucci	2011	1	1	1	0	2	1	0	0	Fair
Aimee S. Browne	2012	1	1	0	1	2	1	1	0	Good
Bárbara Herranz-Blanco	2023	1	0	0	1	2	1	1	0	Fair
Andrea Giannini	2010	1	0	0	0	2	1	0	0	Poor
Sri Ratna Dwiningsih	2022	1	1	0	1	2	1	1	0	Good
Shaojie Ding	2017	1	1	0	1	2	1	1	0	Good
Yu	2023	1	1	0	1	2	1	1	0	Good
Wessels	2016	1	1	0	0	2	1	0	1	Fair
Stefani	2019	1	1	1	0	2	1	0	0	Fair
Perricos	2018	1	1	0	1	2	1	1	0	Good
Liang	2020	1	1	0	0	1	1	0	0	Poor

### Meta-analysis results

**Fig. 2:**
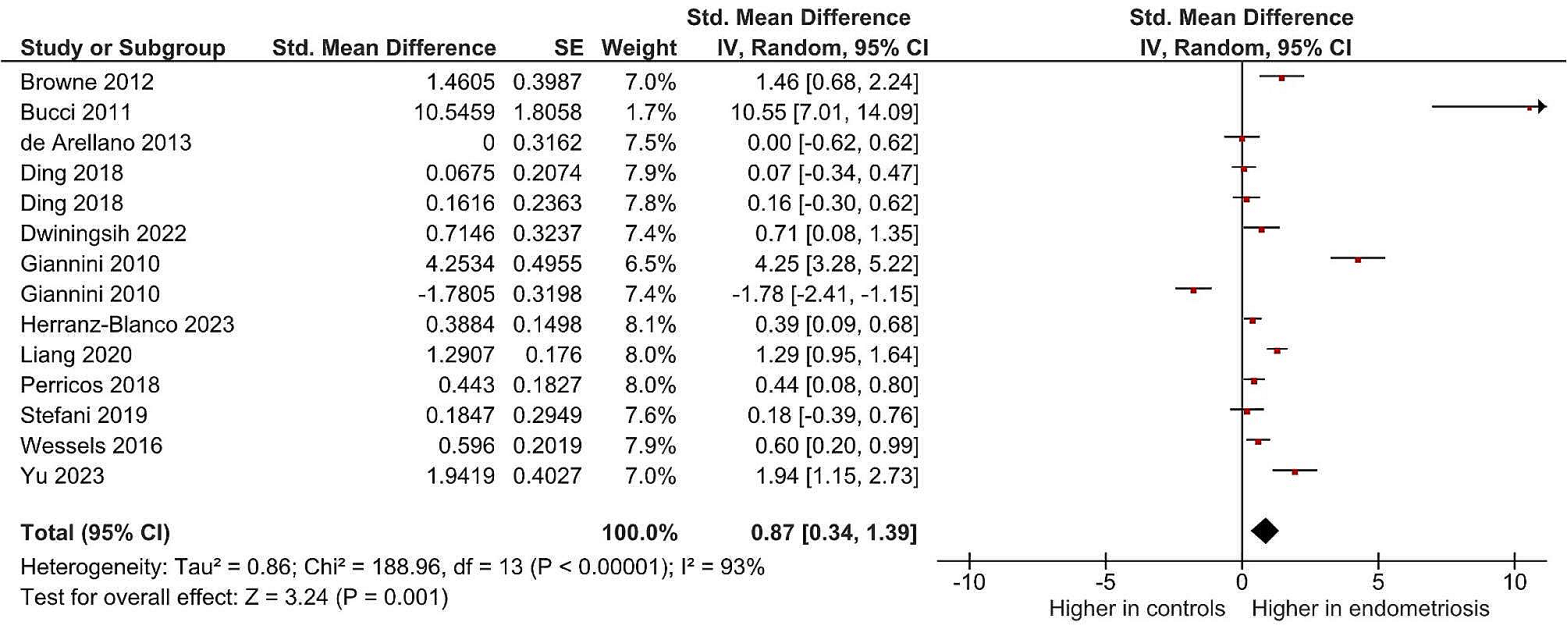
Results of meta-analysis for the level of Brain-Derived Neurotrophic Factor (BDNF) levels in patients with endometriosis

The meta-analysis of the included studies revealed a significant association between BDNF levels and endometriosis. The pooled standardized mean difference (SMD) of BDNF levels between women with endometriosis and controls was 0.87 (95% confidence interval [CI] 0.34 to 1.39, *p* = 0.001; I2 = 93%), indicating higher BDNF levels in women with endometriosis compared to controls. The forest plot depicting the individual study results and the overall pooled effect is presented in Fig. 2.

### Publication bias

**Fig. 3:**
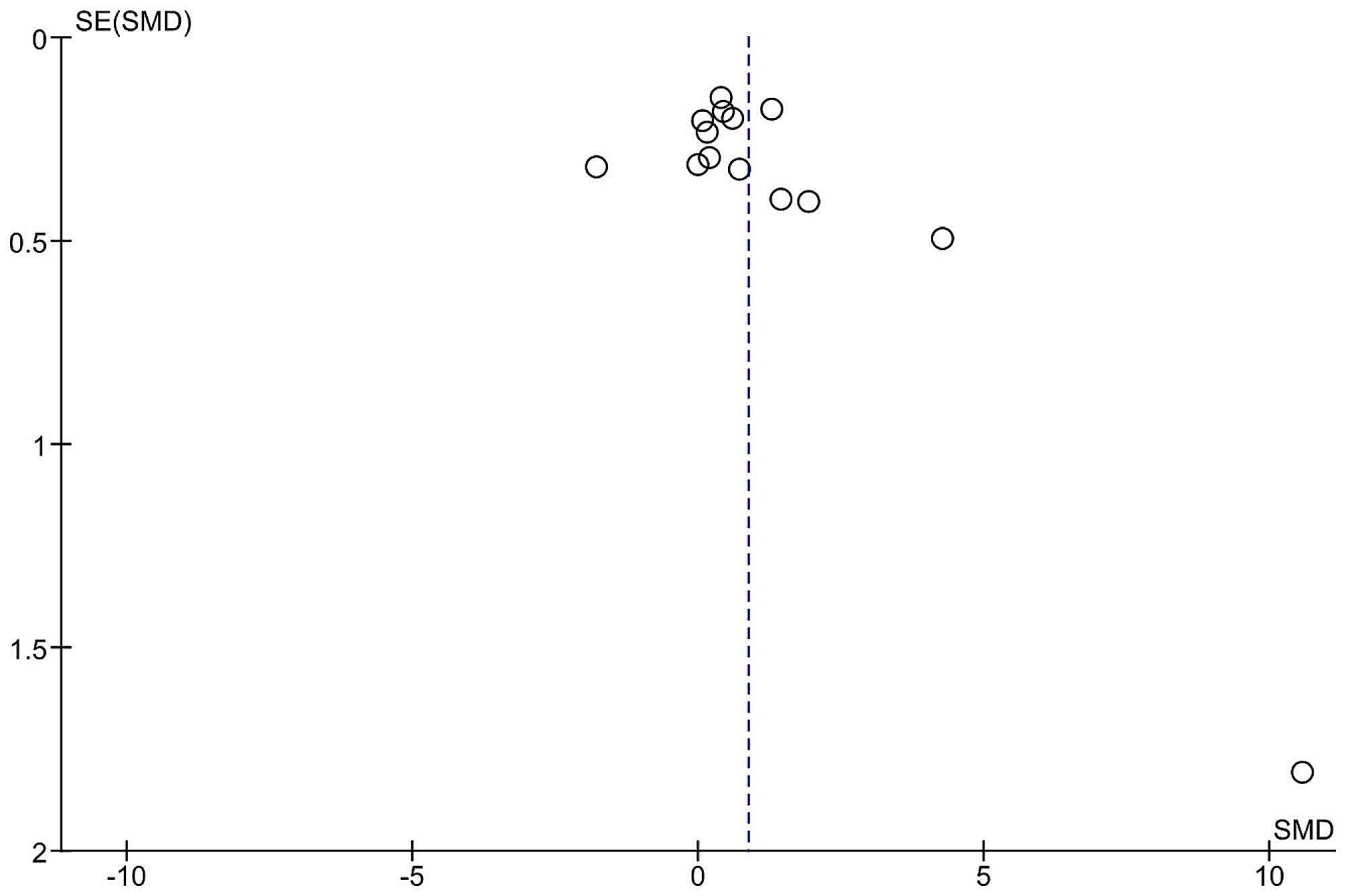
Funnel plot

Publication bias was assessed using funnel plots and Egger’s test. The funnel plot appeared symmetrical, indicating no significant publication bias. Egger’s test also confirmed the absence of publication bias (*p* = 0.15) (Fig. 3).

### Sensitivity analysis

**Fig. 4:**
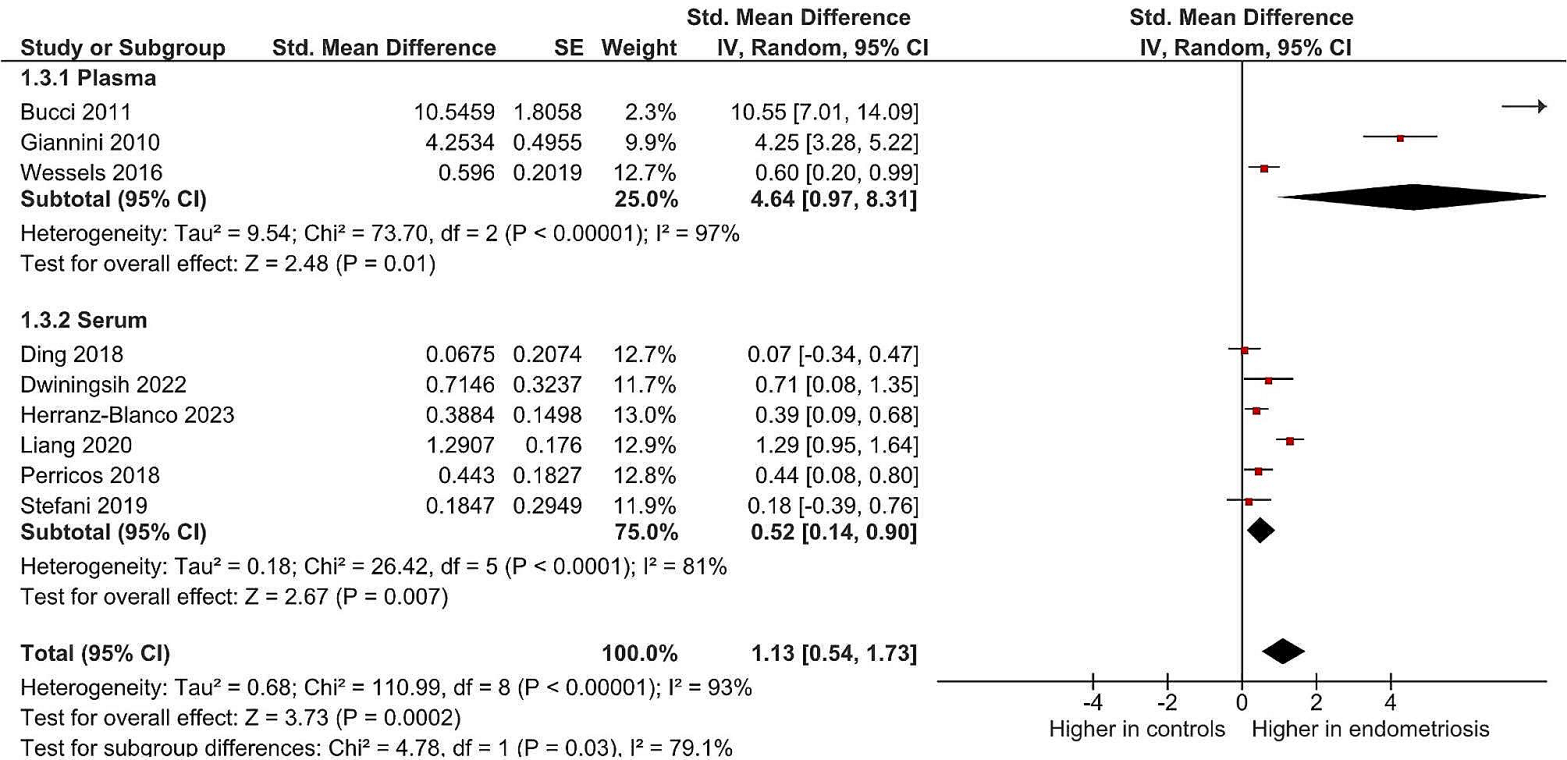
Results of sensitivity analysis

A sensitivity analysis was conducted by studies that assessed blood levels of BDNF. The results showed that blood levels of BDNF are significantly higher in endometriosis patients (SMD: 1.13 95% CI 0.54 to 1.73, *p* = 0.0002; I2 = 93%) (Fig. 4).

## Discussion

The result of the present systematic review and meta-analysis indicates that BDNF levels significantly increase in patients diagnosed with endometriosis compared to healthy controls. The result of the sensitive analysis showed a significant increase in BDNF levels in both plasma and serum in endometriosis.

Evidence showed that BDNF level varies during a healthy menstrual cycle, and it is reported that BDNF significantly increases during the Luteal phase in comparison with the follicular phase [19]. It is also mentioned that BDNF is significantly lower in Amenorrhoeic subjects, as well as postmenopausal women [19]. Taken together, all this evidence shows that estradiol and progesterone might have an impact on BDNF circulation, and also literature showed a positive correlation between BDNF and E (2) and progesterone in fertile women [19].

Results of a study done by Bucci et al. revealed a significantly higher level of estradiol and progesterone among patients with stage 1 and 2 endometriosis compared to healthy controls [12]. It can therefore be assumed that BDNF can increase in patients diagnosed with endometriosis.

This study produced results that corroborate the findings of a great deal of the previous work in this field. Giannini et al. found that the level of BDNF in plasma was significantly higher in comparison with healthy controls in the follicular phase, also the results of a study done by Browne et al. are consistent with Giannini et al. study and showed a higher level of BDNF in patients diagnosed with endometriosis [9, 14]. However, the findings of the Ding et al. and De Arellano et al. studies do not support the results of the studies mentioned earlier, they revealed no significant difference between healthy controls and women with endometriosis in the level of BDNF [10, 13]. A systematic review done by Chow et al. indicates that Pro-BDNF is expressed in the endometrium, and BDNF expression in the endometrium is significantly higher in patients with endometriosis [20]. These findings may be a possible explanation for the results of Browne et al. study which showed that although BDNF concentration was higher in women with endometriosis, three months after surgical removal of endometriotic lesions, no difference was found in the level of BDNF between healthy controls and women with endometriosis [9]. Wessels et al. compared BDNF levels in patients who received treatment for endometriosis with patients who did not, the results showed a significantly decreased BDNF level in the treated group [6]. Although BDNF was significantly higher in endometriosis compared with healthy controls, no significant changes were reported between different stages of endometriosis [6, 11]. However, BDNF expression in eutopic endometrium is positively correlated with stages of endometriosis [7]. A study done by Rocha et al. showed that although BDNF is higher in plasma among patients with ovarian endometrioma and can be used as a diagnostic marker, it is not helpful for the diagnosis of other forms of endometriosis including peritoneal or deep infiltrating endometriosis [21].

BDNF expression plays an essential role in female reproductivity by affecting placental function, oocyte maturation, embryo development, follicle development, and oogenesis, therefore dysregulation of BDNF can lead to several serious complications in women such as endometriosis, intra-uterine growth restriction (IUGR), preeclampsia and cancers [20]. A positive correlation is reported between estrogen and BDNF, and the interaction of inflammatory factors [Interleukin-1β (IL-1β)] and estradiol (E2) with their receptors leads to increased extracellular signal-regulated kinase 1/2 (ERK1/2) expression, within transcription factor phosphorylation, cAMP response element binding protein (CREB) causes synthesis of BDNF in the endometrium [10]. Capillary blood vessels formed around endometriosis tissue would help this increased amount of BDNF reach the peripheral circulation.

To the best of our knowledge, the present systematic review and meta-analysis is the very first study that investigates the level of BDNF in patients with endometriosis and evaluates the diagnostic value of BDNF in endometriosis. Also, our study has extended the results of previous studies on this topic by including 12 studies. Additionally, in our sensitive analysis, we have compared BDNF levels in serum and plasma separately, which can lead to a better vision for utilizing the BDNF as a novel biomarker for endometriosis. However, with a small sample size, caution must be applied, as findings might not be transferable to all the patients who are diagnosed with endometriosis. Only 50% of the included studies have evaluated the level of BDNF in either serum or plasma, since it is easier for both health workers and patients to evaluate BDNF in blood samples, more studies are required to investigate BDNF levels in blood.

Number of limitations should be considered for current study. Several confounding factors are able to make changes in BDNF level in individuals such as socioeconomic status which can lead to escalating rate of depression, different type of mental disorders and administration of number of medicines including Analgesics. [22] Included studies in our meta-analysis have not considered mentioned factor in their participants, therefore evaluated BDNF level in these studies can be effected by confounding factors. Other limitation for our study is number od included articles and participants, for considering BDNF as a diagnostic value for endometriosis, more studies should be included and determined.

Considerably more work will need to be done to determine the correlation between BDNF level and endometriosis and to evaluate the diagnostic value of BDNF. These would help health workers with earlier diagnosis, more efficient treatment, and controlling the adverse effect of endometriosis such as pain and infertility. As mentioned earlier, since BDNF increases in both serum and plasma, it can be utilized as an accessible, fast, non-invasive, and inexpensive method for not only diagnosis but also evaluating the severity and treatment respond in women with endometriosis.

In conclusion, our study revealed that BDNF level is significantly higher in patients with endometriosis compared to healthy control. Further investigation and experimentation into the correlation between BDNF and endometriosis is strongly recommended.

### Electronic supplementary material

Below is the link to the electronic supplementary material.

Supplementary Material 1

## Data Availability

All data generated or analyzed during this study are included in this published article [and its supplementary information files].
